# Increasing access to modern contraceptives: the potential role of community solidarity through altruistic contributions

**DOI:** 10.1186/1475-9276-11-34

**Published:** 2012-07-06

**Authors:** Obinna E Onwujekwe, Chinwe Ogbonna, Nkoli Uguru, Benjamin SC Uzochukwu, Agathe Lawson, Bannet Ndyanabangi

**Affiliations:** 1 Health Policy Research Group, Department of Pharmacology and Therapeutics, College of Medicine, University of Nigeria, Enugu, Nigeria; 2 Department of Health Administration and Management, College of Medicine, University of Nigeria, Enugu, Nigeria; 3 United Nations Population Fund (UNFPA), Abuja, Nigeria; 4 Department of Preventive Dentistry, College of Medicine, University of Nigeria, Enugu, Nigeria; 5 Department of Community Medicine, College of Medicine, University of Nigeria, Enugu, Nigeria

**Keywords:** Contraceptives, Altruism, Altruistic WTP, Willingness to pay, Community Solidarity

## Abstract

**Background:**

There is an urgent need for universal access to modern contraceptives in Nigeria, to facilitate the achievement of the Millennium Development Goals and other national goals. This study provides information on the potential role of community solidarity in increasing access to contraceptives for the most-poor people through exploration of the role of altruism by determining level of altruistic willingness to pay (WTP) for modern contraceptives across different geographic contexts in Nigeria.

**Methods:**

It was a cross-sectional national survey which took place in six states spread across the six-geopolitical zones of the country. In each state, an urban and a rural area were selected for the study, giving a total of 6 urban and 6 rural sites. A pre-tested interviewer-administered questionnaire was used to collect information from at least 720 randomly selected householders from each state. The targeted respondent in a household was a female primary care giver of child bearing age (usually the wives), or in her absence, another female household member of child bearing age. A scenario on altruistic WTP was presented before the value was elicited using a binary with open-ended follow-up question format. Test of validity of elicited altruistic WTP was undertaken using Tobit regression.

**Findings:**

More than 50 % of the respondents across all the states were willing to contribute some money so that the very poor would be provided with modern contraceptives. The average amount of money that people were willing to contribute annually was 650 Naira (US$4.5). Mean altruistic WTP differed across SES quintiles and urban-rural divide (p < .01). Multiple regression analysis showed that age was negatively related to altruistic WTP (p < 0.05). However, years of schooling, being employed by government or being a big business person, prior experience of paying for contraceptives and socioeconomic status had statistically significant effects on altruistic WTP (p < 0.05).

**Conclusion:**

There is room for community solidarity to ensure that the very poor benefit from modern contraceptives and assure universal coverage with modern contraceptives. The factors that determine altruistic WTP should be harnessed to ensure that altruistic contributions are actually made. The challenge will be how to collect and pool the altruistic contributions for purchasing and delivering modern contraceptives to the most-poor, within the context of community financing.

## Introduction

In recognition of the urgent need for universal access to reproductive health services in Nigeria, there is a need to scale-up the provision and utilisation of contraceptives in the country for improved reproductive health outcomes of the citizens. Hence, policy makers and programme managers in the area of family planning require available evidence-based information that will enable them make appropriate decisions on scaling up access to modern contraceptives in Nigeria. The 2008 NDHS in Nigeria found that only 10% of married women of reproductive age use modern contraceptives, which is lower than the current Sub Saharan African average of 17% [1].

The relationship between contraceptive prevalence rates and maternal mortality is evident in the fact that countries with low contraceptive prevalence rates also have very high mortality ratios [2]. Nigeria has one of the highest maternal mortality ratio in Sub-Saharan Africa, and ranks as the country with the second highest number of maternal deaths in the world [3]. Illegal and unsafe abortions contribute 20%–40% of about 60,000 maternal deaths that occur yearly in the country [4]. One of the causes of the high mortality rate in Nigeria can be attributed to the high fertility rate in the country especially in the rural areas and northern parts of the country, which has in turn been associated with the low rate of contraceptive use [1].

The use of modern contraceptive methods in sexually active people, translates into the prevention of unwanted pregnancy and subsequent abortions emerging thereof, which will ultimately lead to a significant reduction in maternal mortality [5]. In a previous study it was observed that 14% of Nigerian women have tried to have an abortion while about 10% have ended up with unwanted pregnancies suggesting that up to 760,000 induced abortions could occur annually [6]. The rate of induced abortions can therefore be said to be a good indicator of the current state of family planning in any country [7]. Research in Nigeria indicates that more than 60% of women with an unplanned pregnancy are not using any form of contraception [8].This low prevalence in the use of modern contraceptives have been attributed to various factors like lack of awareness or access to these contraceptives, cultural and religious factors, the myth of side effects and the weak political will by Government at all levels to provide predictable funding for family planning (FP) programmes on a larger scale, inclusive of community oriented approaches and enhanced awareness creation [5].

Limited government and donor resources, as well as the need to meet ever-increasing contraceptive demand, therefore indicates that reproductive health programmes should explore new ways to increase and sustain availability of financial resources[5]. This could be by the adoption of a whole market approach, which will use user fees from the private sector to generate revenue needed or accrue same through subsidies and or premium from risk pooling mechanisms in order to expand services while at the same time trying to make it affordable for the poor. This however is an onerous task because, identifying a clients’ ability to pay for family planning services is difficult [5].

A possible strategy for increasing access to contraceptives especially for the poor and vulnerable group is through altruistic community solidarity, whereby the economically better-off households or individuals contribute money so that contraceptives could be procured and provided to the poor. This practice is common in many spheres of life in Nigeria and could be potentially harnessed for increasing access to modern contraceptives [9]. The strategy would be to cultivate informal communities that have sources of revenue and relevant populations in relevant quintiles to unleash their altruistic nature and contribute money for improved access of vulnerable community members to modern contraceptives.

Altruism will play a role in ensuring that contraceptives are available to the poor. It is possible that the well to do or not so poor people living in a community may be willing to contribute some money for the most poor to have access to modern contraceptive measures, especially if the better-off people are convinced of the benefits of these contraceptive measures [9].

Altruistic behaviour, a form of pro-social behaviour is defined as voluntary, intentional behaviour that benefits another and is not motivated by the expectation of external rewards or avoidance of externally produced punishments [10]. It is “devotion to the welfare of others, regardless of one’s own welfare” [11]. The concept of altruism is arguably a strategy for maximizing sustainability and survival among a social group which provide important benefits for the group [12]. Altruism is sometimes embarked on because the image of being an altruist or generous person motivates people to be selfless regardless of their current reputations or identity [12]. These are behaviours that could be harnessed to increase coverage of modern contraceptives, especially amongst the poor considering the need to identify alternative sources of funding for family planning programmes being that the erstwhile cost recovery scheme in public health facilities has been removed by government.

The level of altruistic behavior could be elicited using the contingent valuation method, to determine the maximum amount of money that people will be willing to contribute so that poor people in their community can have free access to modern contraceptives [9,13-15]. Hence, altruistic WTP investigates the potential of more capable people to contribute to the poor in their communities [13]. In previous studies it was observed that a greater percentage of the study population were hypothetically willing to pay for the poor in their communities, although albeit a lesser percentage actually made altruistic payments [13].

The paper contributes new knowledge on the levels of altruistic willingness to pay (WTP) for contraceptives delivered through the public sector in Nigeria. It provides information on the maximum amount of money that different population groups resident in different parts of the country are willing to contribute for the poor to benefit from deployment of major contraceptives. Contingent valuation method (CVM) was used to elicit altruistic WTP. CVM is one of the most commonly used valid direct techniques for eliciting willingness to pay [16]. The method involves respondents evaluating, in monetary terms, goods or services with benefits that may not be directly measurable [17].

## Study methodology

### Study area

The study took place in the six-geopolitical regions of the country and hence covered the different contexts found nationally in Nigeria. The Federal Republic of Nigeria has a projected 2010 population of 160, 821, 353 and the country has the largest population in Africa and tenth largest in the world and at the current annual population growth rate of 3.2 percent, the population is expected to double by the year 2030 [1]. This rapid population growth rate can be attributed to the high total fertility rate (TFR) of 5.7, an unmet need for family planning establshied at only 20%, a low contraceptive prevalence rate of 10% for modern methods, while about 23 percent obtain contraception from a public sector facility [1]. The country has 36 states and a federal capital territory (FCT). The states are arranged into six-geoplotical zones: Northeast; Northcentral; Northwest; Southeast; Southsouth; and Southwest zones. The FCT is technically in the Northcentral zone. The study locations were the Federal Capital Territory (FCT or Abuja) (Northcentral zone), Kano state (Northwest zone), Lagos state (Northwest zone), Enugu State (Northest zone), Adamawa state (Northeast sone) and Rivers state (Southsouth zone). In each state, an urban and a rural area were selected for the study. Hence, there were 6 urban and 6 rural sites from the six states.

#### Conceptual framework

Altruistic WTP was elicited using the contingent valuation method. Determining WTP through the CVM is increasingly being used to generate information on the benefits of, and demand for, health care programmes. WTP is the maximum amount of income an individual is willing to give up in order to ensure that a proposed service or good is available [18]. The WTP could be for the availability of a resource to the individual (own use), for others e.g. the poor people (altruism) or needed by others or the individual in the future (option or non-use) or a combination of any of these measures [13].

#### Study design and study tools

It was a cross-sectional quantitative study. A pre-tested interviewer-administered questionnaire was used to collect information from randomly selected householders. Altruistic willingness to pay (WTP) of the respondents for major modern contraceptives available in the public sector was elicited. The contraceptives were Oral contraceptive pills (OCP), injectables, male and female condoms, intra-uterine devices (IUDs) and implants. The questionnaire was also used to collect data on socio-economic and demographic characteristics of households as well as previous experience with contraceptives.

The questionnaire was administered to respondents selected by simple random sampling from a sample frame of households. The targeted respondent in a household was the main beneficiary of use of modern contraceptives for child spacing and this was a female primary care giver of child bearing age (usually the wives), or in her absence, another female household members of child bearing age and in her absence after repeated visits, the male head of household. However, all attempts were made to ensure that the respondent was the wife. Adequate sample size was determined, using a power of 80%, confidence level of 95% confidence level and utilization rate of contraceptives of 10%. This gave a minimum sample size of 350 per urban and rural site. However, in order to control for refusals and incomplete questionnaire, the number of respondents to be interviewed was increased to 385 per site, yielding 770 per state.

A scenario on altruistic WTP was presented before the value was elicited using a dichotomous choice (binary) with an open-ended follow-up method. The WTP of the respondents for their own use of the different contraceptives was elicited before altruistic WTP was elicited. The WTP scenarios for the different contraceptives explained their uses, pros and cons before WTP was elicited. Hence, the respondents had good level of different contraceptives before they were asked to state their maximum altruistic WTP amounts. It was decided to use 500 Naira as the starting bid because it was close to the monthly capitation payment of the formal sector programme of the National Health Insurance Scheme. Hence, it was an amont of money that respondents could relate to.

The questions asked were:

1. Are you willing to contribute 500 Naira per year so that contraceptives could be procured and given to the poorest people in your community that need them? [] 1 = yes 0 = no (no matter the answer, go to 2).

2. What is the maximum amount of money that you are willing to contribute yearly so that contraceptives would be procured for some of the needy poorest people in your community? [] Naira.

"As you may know, there are some people who are too poor to pay any money for contraceptives, but they really need the contraceptives so that they can improve child spacing, their health status and productivity."

## Data analysis

The average altruistic WTP estimates were computed in addition to number of people that gave affirmative answers. The validity of the elicited altruistic WTP was undertaken using Tobit regression. This is because the final open-ended questions ended up generating continuous limited dependent WTP amounts, which were limited at zero. Tobit estimation refers to regression models in which the range of dependent variable is constrained in some way [19]. If the dependent variable is limited in some way, ordinary least squares (OLS) estimates are biased even asymptotically [20]. OLS in this case fails to account for the qualitative differences between the limit observations (those with zero WTP) and the non-limit observations [21]. Omitting the limit observations creates bias. Ignoring them will be throwing away information, but including them as though they were ordinary observations also creates bias [20]. It was posited that if the zero observations reflect genuine WTP values of zero, the Tobit model is appropriate for the estimation [21]. **In** the Tobit regression, a logarithm + 1 (log + 1) transformation was used for altruistic WTP estimates starting from 0, to ensure zeros were not treated as missing values after log transformation [20-22]. There was a check for evidence of multicollinearity amongst the independent variables.

The independent variables were: the socio-economic status (SES) of the respondent; the geographic area of residence (1 = urban or 0 = rural); status of the respondent in the household (0 = wide, 1 = adult female representative and 2 = male head of household); the number of household residents; their sex (0 = female, 1 = male); age (years); the highest educational level attained (measured as a dummy variable 0 and1, with 1 representing the highest level attained); number of years of schooling; the occupational group (being unemployed was the base variable); the marital status (being a widow was the base variable); and whether the respondent had previously paid for any contraceptive (1 = paid, 0 = not paid). A cut-off of <0.10 was used to decide on whether variables were statistically significantly related to WTP.

The data was also examined for relationships between socio-economic status (SES) and geographic location with altruistic WTP. For specifically analyzing the socio-economic equity implications of the data from the consumers, an asset-based socio-economic status (SES) index was created using principal components analysis [23]. Information on household ownership of radio set, bicycle, television set, motorcycle, fridge, as well as per capita weekly food value were the variables in the SES index. The first principal component was used to derive weights for the SES index. The SES index was used to divide the households into quintile and chi-square analysis used to determine the statistical significance of the differentiation of WTP into SES quartiles. Note US$1 = 145 Naira.

### Ethical considerations

All respondents gave informed consent before proceeding with a WTP interview. The study participant had the right to leave at any time without penalty. The respondents were given an opportunity to ask questions and be fully informed before signing the consent form. Consequently, the interviewee’s consent to participate in the study was demonstrated by signing the one original and one copy of the consent form. In addition to the consent form, ethical clearance for the study was given by the EthicsCommittee of University of Nigeria.

## Findings

### Section 1: Socio-demographic distribution of the respondents in the six states

The response rate in the six states was more than 95% and a total of 4517 questionnaires were analysed. The number of analysable questionnaires ranged from 726 in Lagos state to 776 in Rivers state. Most of the respondents in the six states were either the wives or adult female household representative and were married (Table 1). The average age of the respondents was 31 years. The number of household residents (number of people that are resident in a household) ranged from 4.9 in Lagos state to 8.3 in Kano state. Most of the people also had some form of formal education and the commonest completed educational levels were senior secondary school (SSS), followed by universities or polytechnics and the average number of years that the respondents spent in school was 11 years. Table 1 also shows that the most common occupation of the respondentswas petty trading/artisan, followed closely by unemployment. The average weekly food expenditure ranged from 2832 Naira (US$ 18.9) in Enugu to 9187 Naira (US$61.2) in Rivers state, with the group average as about 6000 Naira (US$40.0). The same pattern was seen for per capita weekly food expenditure, where the group mean value was 1156 Naira (US$7.7).

**Table 1:** Socio-demographic distribution of the respondents in the six states

**Variable**	**Abuja N =771 n(%)**	**Adamawa N =728 n(%)**	**Enugu N = 769 n(%)**	**Kano N = 747 n(%)**	**Lagos N = 726 n(%)**	**Rivers N = 776 n(%)**	**Combined N = 4517 n(%)**
Wife	405 (52)	664 (91)	658 (86)	460 (62)	537 (74)	584 (75)	3308 (73)
Adult female household rep	265 (34)	42(6)	106 (14)	51 (7)	176 (24)	177 (23)	817 (18)
Female respondents	671 (87)	708 (974)	763 (99)	509 (68)	711 (98)	761 (98)	4123 (91)
Age mean (SD)	30.9 (8.0)	31.6 (7.6)	31.0 (7.1)	32.1 (7.0)	31.0 (6.7)	31.9 (6.2)	31.4 (7.1)
No of household members mean (SD)	5.4 (2.2)	7.2 (4.9)	5.1 (2.2)	8.3 (4.8)	4.9 (5.3)	5.3 (2.1)	6.0 (3.5)
Attended School	742 (96)	460 (63)	730 (94)	581 (77)	710 (98)	762 (98)	3985 (88)
Highest level of education							
Primary School	54(7)	125(27)	152(21)	116(20)	60(8)	38(5)	545(14)
Junior Secondary School	40(5)	57(12)	66(9)	83(3)	23(3)	32(4)	301(8)
Senior Secondary school	264(36)	137(30)	275(38)	219(38)	299(42)	393(52)	1587(40)
Teacher training college	21(3)	8(2)	26(4)	31(4)	14(2)	16(2)	116(3)
College of Education	34(5)	71(15)	25(3)	47(8)	24(3)	82(11)	283(7)
University of Polytechnic	310 (40)	47 (6.4)	182 (23.7)	71(9.3)	259 (35.7)	186 (23.9)	1055 (23.4)
Others	18(2)	14(3)	4(.5)	97(17)	28(4)	17(2)	178(5)
Years in school mean (SD)	12.7 (4.3)	7.0 (6.2)	11.0 (4.7)	7.8 (6.4)	12.6 (3.6)	12.8 (3.8)	10.7 (5.5)
Employment status of the respondent							
Unemployed	178(16)	321(29)	175(16)	155(14)	167(15)	95(9)	1091(25)
Subsistence farmer/herd keeper	19(3)	25(10)	74(2)	13(2)	1(.1)	53(7)	185(4)
Petty trader/artisan	136(18)	245(34)	237(31)	240(34)	93(29)	219(29)	1170(26)
Govt worker	127(17)	97(13)	99(13)	89(13)	25(3)	109(14)	546(12)
Private sector employee	90(12)	18(3)	64(8)	15(2)	48(7)	157(21)	392(9)
Big biz/self employed	158(21)	14(2)	95(12)	70(10)	327(45)	77(10)	741(17)
Others	77(10)	7(1)	39(5)	129(18)	58(8)	50(7)	360(8)
Marital status: Married	487(63)	652(90)	652(85)	664(94)	554(76)	589(77)	3598(81)
Food value mean (SD)	5269.8 (3065.3)	6398.0 (4413.0)	2831.8 (1226.8)	7485.8 (6268.7)	4818.8 (3852.6)	9186.6 (5130.5)	6003.5 (4748.1)
Per capita food value mean (SD)	1110.9 (913.6)	1021.2 (618.8)	656.7 (464.6)	1098.5 (1244.6)	1151.4 (1803.6)	1883.0 (1227.0)	1156.2 (1189.2)

### Altruistic willingness to Pay

More than 50% of the respondents were willing to contribute the starting-bid amount so that the poor could have access to modern contraceptives (Table 2). The average annual altruistic WTP ranged from US$2.4 in Enugu state to US$6.4 in Rivers state. The differences in altruistic WTP across the states were statistically significant (p < .0.01).

**Table 2:** Levels and average altruistic willingness to pay

**Variable**	**Abuja n (%)**	**Adamawa n (%)**	**Enugu n (%)**	**Kano n (%)**	**Lagos n (%)**	**Rivers n (%)**	**Combined n (%)**	***X*2 (p-value)**
Whether willing to contribute: n(%)	478 (62)	514 (71)	384 (50)	391(52)	562 (77)	606(78)	2935 (65)	250.0 (<.001)
Average annual altruistic WTP amount in Naira: mean (SD)	750.7 (1261.0)	696.5 (926.3)	363.6 (374.9)	613.4 (1430.4)	792.1 (1328.3)	955.4 (1082.4)	695.1 (1129.5)	389.1 (<.01)
Average annual altruistic WTP amount (US$)	5.0	4.6	2.4	4.1	5.3	6.4	4.6	

### Reasons that respondents were not willing to pay for altruism

The major reason indicated by respondents for their unwillingness to contribute for altruism was lack of money (Table 3). This was most evident in the responses from all the states though a greater percentage of those living in Enugu stated this (76%) (p < 0.05). Most of the respondents did not consider culture and religion as a barrier to altruism. However, religion was noted as a barrier by 20% and 36% of the respondents in two of the states. Most of the respondents stated that the perception of contraceptives being harmful or their dislike of the contraceptives was not a barrier (p < 0.05).

**Table 3:** Reasons why respondents were not willing to pay for altruism

	**Abuja n(%)**	**Adamawa n(%)**	**Enugu n(%)**	**Kano n(%)**	**Lagos n(%)**	**Rivers n(%)**	***X***^**2**^**(p - value)**
Do not have money	62(38%)	25(22%)	94(76%)	9(7%)	31(42%)	16(16%)	161.633 (<.001)
Do not need it	2(1%)	35(30%)	16(13%)	4(3%)	3(4%)	4(4%)	85.940 (<.001)
It is against my religion to use it	16(10%)	23(20%)	6(5%)	43(36%)	2(3%)	12(12%)	65.114 (<.001)
It is against my culture	1(0.6%)	0 (0)	4(3%)	4(3%)	1(1%)	1(1%)	7.489 (.187)
It is harmful	1(0.6%)	10(9%)	10(8%)	1(0.9%)	0	1(1%)	28.798 (<.001)
I do not like it	2(1%)	9(8%)	14(11%)	22(18%)	2(3%)	10(10%)	29.933 (<.001)
Others	87(55%)	2(2%)	4(3%)	50(41%)	41(55%)	32(32%)	158.191 (<.001)

### Analysis of urban-rural and SES differences in WTP for contraceptives provided by the public sector using the combined data

The average altruistic WTP amount was higher in the urban area (p < .05) (Table 4). However, the rural dwellers were more likely than the urban dwellers to state that they could contribute for altruism, the difference was not statistically significant (p > .05).

**Table 4:** Levels of altruistic willingness to pay by geographic location

**Variable**	**Urban N = 2294**	**Rural N = 2223**	***X*2 (p-value)**
Whether willing to contribute: n (%)	1430 (62.3%)	1505 (67.7%)	0.017(.90)
Average annual altruistic WTP amount: Naira mean (SD)	760.2 (1244.6)	635.0 (1008.4)	17.1 (<.0001)
Average annual altruistic WTP amount in US$	5.1	4.2	

Table 5 shows that altruistic willingness to pay increased with increasing socio-economic status (SES) of the respondents. Hence, the wealthier the respondent is, the higher the altruistic WTP for contraceptives. All the differences in WTP across the SES quintiles were statistically significant (p < .01).

**Table 5:** Levels of altruistic willingness to pay by SES

**Variable**	**Q1 n = 904**	**Q2 n = 904**	**Q3 n = 903**	**Q4 n = 903**	**Q5 n = 903**	**Q1:Q5 ratio**	***X*2 (p-value)**
Whether willing to contribute: n(%)	449 (49.7%)	515 (57.0%)	602 (66.7%)	656 (72.7%)	713 (79.0%)	0.63	220.6 (<.001)
Average altruistic WTP amount in Naira: mean(SD)	385.8 (603.1)	511.7 (841.2)	651.7 (1018.1)	825.3 (1263.2)	1078.2 (1129.5)	0.36	362.9 (<.01)
Average altruistic WTP amount in US$	2.6	3.4	4.4	5.5	7.2		

### Tobit regression

Table 6 shows that age was negatively related to altruistic WTP (p < 0.05). However, years of schooling, being a big business person, prior experience of paying for contraceptives and socioeconomic status had statistically significant effects on altruistic WTP (p < 0.05). Hence, the higher a person’s SES and the more years spent in school, the more the level of altruistic WTP. Table 6 also shows that being employed by government and been a divorcee were positively, although marginally related to altruistic WTP. Females were more altruistic than men. There was no evidence of multicollinearity between the independent variables. The regression was statistically significant.

**Table 6:** Tobit regression analysis showing relationship between altruistic WTP and independent factors

**Independent variables**	**Coefficient**	**Std. Error**	**p-value**
Socio-economic status (SES)	.283	0.338	0.0001
Geographic location of respondent (0 = rural; 1 = urban)	.089	.097	0.356
Status in household	- .054	.170	0.753
Number of household residents	-.003	.014	0.843
Sex (0 = female; 1 = male)	-.633	.351	0.071
Age	-.030	.007	0.0001
Years schooling	.050	.010	0.0001
Subsistence farmer	.012	.244	0.962
Petty trader	.013	.129	0.918
Government worker	.332	.172	0.054
Employed in the private sector	.047	.180	0.795
Doing big business	.325	.148	0.028
Other occupational groups	.130	.180	0.471
Married	-.114	.254	0.653
Divorced	.991	.591	0.093
Separated	.133	.552	0.810
Single	-.251	.310	0.417
Had previously paid for any contraceptive	.799	.107	0.0001

LR chi^2 =^ 286.78 (p = 0.00001).

The study has shown that an appreciable level of altruism for scaling up access to modern contraceptives exists in Nigeria. It was found that many respondents across all the states were willing to contribute some money so that the very poor would be provided with modern contraceptives and the average amount of money that people were willing to contribute annually was 650 Naira (US$4.3). However, the average amounts of money that people were willing to contribute to ensure community solidarity (altruism) differed by states, urban-rural divide and socio-economic status.

The fact that there were high levels of willingness of respondents to contribute varied amounts of money so that the poor can have free access to modern contraceptives shows that there is room for community solidarity to ensure that the very poor also benefit from modern contraceptives. This is in line with the high sense of solidarity and social justice that exist in many Nigerian communities.

The implication of the high level of altruistic WTP is that in risk pooling through community financing mechanisms such as community-based health insurance (CBHI), there could be contributions of premium by the people in higher SES quintiles so that the very poor could be enrolled and benefit from the services. This is especially pertinent as the National Health Insurance Scheme (NHIS) has included family planning services in its benefit package, which will also be reflected in CBHI schemes that the NHIS is rolling out in Nigeria.

Some of the factors that were related to altruistic WTP, especially their direction of influence were expected a priori. Most of them are related to income, knowledge or past experiences effects. The higher SES quintile groups and people involved in big businesses were more willing to pay for altruism than others, which could be seen as purely an income effect, coupled with a feeling of community support by higher SES groups. A previous study showed that income was positively associated with altruism that is the higher one earns the more likely to contribute to altruism [15].

It was also interesting to find that increasing level of education led to increased level of altruism for contraceptives. This could be related to education contributing to increasing level of awareness of usefulness of contraceptives and the need to protect everybody especially the most-poor from unwanted pregnancies. Therefore as educational status increases, improved awareness of benefits of increased universal access to modern contraceptives increases. This finding implies that increasing educational level of people and hence their awareness about the benefits of modern contraceptives, will increase altruism and community solidarity and lead to increased coverage with modern contraceptives, especially within current efforts to identify alternative funding sources for distribution and operational costs. A previous study showed that educational attainment was significantly associated with altruistic behavior [9].

Also, the finding that age has a negative impact on altruism could point to the fact that at a younger age people are more likely to be willing to pay for the poor because they are still in the working class age group. Hence, it was valid to find that younger people were more willing to make altruistic contributions as they are also expectedly would have being using modern contraceptives and are more aware of the benefits. This assertion is supported by the finding in this study that people that had previously paid for modern contraceptives were more willing to make altruistic contributions compared to people that had not made such payments.

Geographic location of the respondents affected altruism because the urban dwellers who are more likely to have a higher income than those in the rural areas were willing to contribute less altruistically than those in the rural areas. This is not surprising as the rural areas are expected to show more community solidarity because of their homogenous nature than the urban dwellers that are known to be more contextually heterogeneous. However, the fact thatthe Tobit estimation showed that when other variables are taken into consideration, geographic location was not a significant predictor of altruistic WTP does not really negate the possible geographic effects on altruistic WTP.

The finding that females were more willing to make altruistic contributions compared to men is tempered by the fact that most of the respondents were females, hence limiting the direct comparability of altruistic WTP of males and females. Nonetheless, the higher WTP of females could be related to use and perception of personal benefits as most modern contraceptives are intended to protect women from unwanted pregnancies. Hence, although in general, the man is the head of the household and as such is responsible for most charitable giving decisions in the household [24], this may not be case for modern contraceptives where women possibly attach higher benefits that can accrue to the women in the poorest quintile if they are protected from unwanted pregnancies. Nonetheless, it has been shown that females have been observed to be more prone to altruism when the price of giving is expensive and males are more altruistic when the price of giving is low. But on the same budget, there is no difference [24].

The finding that the better-off SES were more WTP altruistically is an income effect, because they probably had spare money to spare to help the most-poor compared to the worse-off SES that may not have any money left. Altruism also depends on how important the services are to the targeted population. Those that have been buying modern contraceptives are more prone to altruistic payment for modern contraceptives because they have already used it and have seen the benefits of contraception and are able to decide its level of importance which will influence their decision to pay for others. Hence, the finding that people that had spent some money on contraceptives were more altruistic than others is not surprising.

It was found that lack of money was cited as the major reason for negative altruism amongst some of the respondents. This is related to the income effect already discussed where altruistic WTP increases as SES increases. It was also found that culture and religion did not constrain altruism and this could be attributed to the fact that altruism is seen to be inherent in most humans especially with the concept of kin selection where a person becomes altruistic if ones kin, relations or friends is in need [25]. Also, it has been argued that cultures of less industrialized societies are most altruistic because the people tend to live in large families and everybody contributes to the welfare of the family [26].

A study limitation is that a test of criterion validity to determine whether people will actually contribute the altruistic WTP amounts that they stated when asked to do so was not undertaken in the study. However, a previous study in southeast Nigeria showed that there was a strong correlation between stated and actual altruistic WTP for bed-nets [14]. The scenario for altruistic WTP is not detailed and provided information on how contributions will be collected and managed to the respondents. Also, the study focused mainly on the female sex and so would not be representative of the opinion of the males and thus cannot be generalised to the whole population. It is also possible that potential effects could have limited sample calculation and for data analysis.

This study has generated many areas for future studies. For instance, future research should explore the level that the broader society will be able to make altruistic contributions, instead of just women. Another area of future work will include testing the criterion validity of stated altruistic WTP by determining whether people will actually contribute the amounts of money that they stated and further examining gender and age differences in altruistic WTP. Also, future studies could explore how altruistic contributions would be collected and used as part of community-based health insurance schemes. However, information will be required to decide whether the average cost of collecting altruistic contributions is less than the average WTP, so as to determine whether the collection of such contributions are an efficient means of increasing access to modern contraceptives.

All in all, the finding of positive sense of altruism for provision of modern contraceptives implies that community solidarity should be possibly encouraged by programme managers in reproductive health so the lowest SES quintiles can also benefit from contraceptives. This is especially pertinent as the National Health Insurance Scheme (NHIS) has included family planning services in its benefit package, which will also be reflected in community-based health insurance (CBHI) schemes that the NHIS is rolling out in Nigeria. It will also be useful to involve the whole society in making the altruistic contributions, instead of just women of childbearing age. However, considering that communities are the right holders, their needs for specific family planning methods need to be recognized and the type of contraception included in the benefit package as determined from community consultations on the package of care.

## Competing interests

The authors have no competing interests.

## Authors’ contributions

OO, CO, AL and BN conceived the study, OO and CO participated in data collection and OO performed statistical analysis. NU and BU participated in the design of the study, data collection and analysis. OO drafted the manuscript. All authors read and approved the final manuscript.
